# Post-mortem Computed Tomographic Angiography in Equine Distal Forelimbs: A Feasibility Study

**DOI:** 10.3389/fvets.2022.868390

**Published:** 2022-05-11

**Authors:** Chantal Blaettler, Sabine Kaessmeyer, Silke Grabherr, Christoph Koch, Daniela Schweizer, Elke Van der Vekens

**Affiliations:** ^1^Division of Clinical Radiology, Vetsuisse Faculty, University of Bern, Bern, Switzerland; ^2^Division of Veterinary Anatomy, Vetsuisse Faculty, University of Bern, Bern, Switzerland; ^3^Centre Universitaire Roman (Lausanne-Geneva), University Centre of Legal Medicine (CURML), Lausanne, Switzerland; ^4^Swiss Institute of Equine Medicine, Department of Clinical Veterinary Sciences, Vetsuisse Faculty, University of Bern, Bern, Switzerland

**Keywords:** PMCTA, horse, perfusion, cadaver, vascular anatomy

## Abstract

In-depth understanding of pathophysiological processes occurring in the vasculature of the equine distal limb is of great importance to improve both diagnostic and therapeutic approaches to diseases. To gain further insights, a model allowing high-resolution 3D-visualization of the vasculature is necessary. This pilot study evaluated the feasibility of restoring vascular perfusion in frozen-thawed distal equine cadaver limbs without prior preparation using computer tomographic imaging (CT). Five frozen-thawed, radiographically normal forelimbs were perfused with a lipophilic contrast agent through the median artery and radial vein in three phases (arterial, venous, and arterial-venous combined (AVC) dynamic). For comparison, one additional limb was perfused with a hydrosoluble contrast agent. The CT-studies (16-slice MDCT, 140 kV, 200 mA, 2 mm slice thickness, 1 mm increment, pitch 0.688) were evaluated at 11 specified regions for visualization of the vasculature and presence of artifacts or anatomic variations. The protocol used in this study proved to be feasible and provided good visualization (93.1%) of vasculature with low rates of artifacts. During the different phases, vascular visualization was similar, but while filling defects decreased in the later phases, extravasation worsened in the 2 limbs where it was observed. Subjectively, the best quality of angiographic images was achieved during the AVC dynamic phase. Perfusion with hydrosoluble contrast resulted in significantly lower vascular visualization (74.0%) and higher artifact rates. This study shows that reperfusion of frozen-thawed equine distal limbs with a lipophilic contrast agent allows for high-quality 3D-visualization of the vasculature and may serve as a model for *in situ* vascular evaluation in the future.

## Introduction

The vascular system of the equine distal limb plays a fundamental role under physiological and pathological circumstances (1, 2). This is specifically reported in diseases such as laminitis, navicular disease, tendon lacerations and impaired wound healing. To advance the research regarding both pathomechanisms of as well as diagnostic and therapeutic approaches to these diseases, there is a need for high-resolution, 3D-visualization and quantification of the vasculature (3, 4). Angiography of the equine distal limb is currently described using radiography (5, 6), fluoroscopy (7, 8), computed tomography (CT) (9–11), and magnetic resonance imaging (MRI) (12). CT- and MRI-angiography overcome the inherent limitations of 2D-imaging, but there are currently no protocols optimized to show different vascular regions and microvasculature. In addition, these techniques need to be performed in live animals and the latter techniques often require general anesthesia.

An equine cadaver model for distal limb angiography would represent a low-cost alternative or addition to *in vivo* and tissue-based models, allowing *in situ* visualization and evaluation of the vasculature. Such a concept is in line with both the 3R principle of animal experiments (Replace, Reduce, Refine) and the ALARA rules of radiation safety (As Low As Reasonably Achievable). Up to now post-mortem angiography of equine limbs has only been performed in limbs collected immediately after death, combined with prior preparation of the vasculature mostly by systemic and/or local administration of heparinized solutions (7, 13–16). This poses significant limitations to the use of equine cadaver limbs as models for vascular research questions. Apart from being time- and resource-consuming, the immediate post-mortem collection and preparation of limbs also limits the choice to horses with a plannable time of death (i.e., experimental animals).

In human forensic medicine, post-mortem angiography is performed regularly using oily perfusates and contrast agents as proposed and optimized by Grabherr and colleagues (17–19). Lipophilic solutions have proven to be better suited in post-mortem settings than hydrosoluble solutions, as they remain intravascular despite the increasing permeability of the vasculature after death and they are able to flush out blood clots (17, 20). One of the most commonly used lipophilic contrast agents, Angiofil^®^, is based on esters of poly-iodinated linseed oil, which is known to be retained in the vasculature for a long time by creating a barrier to the aqueous milieu (20, 21). Angiofil^®^ has been specifically created for post-mortem angiography and is typically diluted to modify the viscosity of the final liquid (22), as that directly determines the degree of microembolization and thus visualization of smaller caliber vessels (17, 20).

Thus, the first aim of this study was to investigate the feasibility of restoring vascular flow in frozen-thawed equine distal limbs without any prior preparation, using a procedure adapted from the human multi-phase post-mortem CT-angiography (MPMCTA) protocol (19). The second and third aims were to report which vessels could be reperfused using this technique and to describe the observed artifacts. We hypothesized that this protocol would allow reperfusion and visualization of the equine distal limb vasculature down to the level of the terminal arch and perforating branches without the occurrence of major artifacts, such as extravasation or filling defects.

## Materials and Equipment

One left and four right forelimbs, disarticulated at the level of the middle carpal joint, were collected from adult horses slaughtered at a local abattoir and frozen at −20°C for ~2–5 weeks. One additional left forelimb (limb 2) was acquired from the equine hospital and underwent two freezing-thawing-cycles due to reasons unrelated to the study, being thawed for 5 days between both cycles. The owner of the horse signed an informed consent form permitting the use of tissues and images for research purposes. To exclude limbs with obvious signs of pathologies, orthogonal radiographs of the fetlock and foot were made and evaluated by a board-certified veterinary radiologist (EVdV) prior to the perfusion.

A lipophilic contrast agent [Angiofil^®^, Fumedica AG, Muri, Switzerland] was diluted in paraffinum perliquidum [Huile de paraffin, Ideal Chimic SA, Carouge, Switzerland] to create a 6% solution (19). Paraffinum perliquidum was chosen instead of paraffinum liquidum because of its lower viscosity, which allows for a better perfusion of small caliber vessels (22).

One limb (limb 6) was perfused with diluted hydrosoluble iodinated contrast medium (37.5 mg/mL iodine in saline) [Accupaque^TM^, 300 mgI/mL, GE Healthcare AG, Opfikon, Switzerland]. In all cases perfusion was performed through stainless steel cannulas [Veterinär-Kanülen, Unimed SA, Lausanne, Switzerland] using a roller pump [Exacta Vet, Schoch Electronics AG, Otelfingen, Switzerland]. Images were acquired using a 16 slice CT-scanner [Brilliance 16 slice, Philips, Eindhoven, The Netherlands], reconstructed using both a bone and soft tissue kernel, sent to the PACS system [IMPAXEEServer_Rad, Agfa HealthCare, Mortsel, Belgium] in DICOM format for storage, and evaluated using a DICOM-reader [IMPAX EE R20, Agfa HealthCare, Mortsel, Belgium].

## Methods

**Figure 1:**
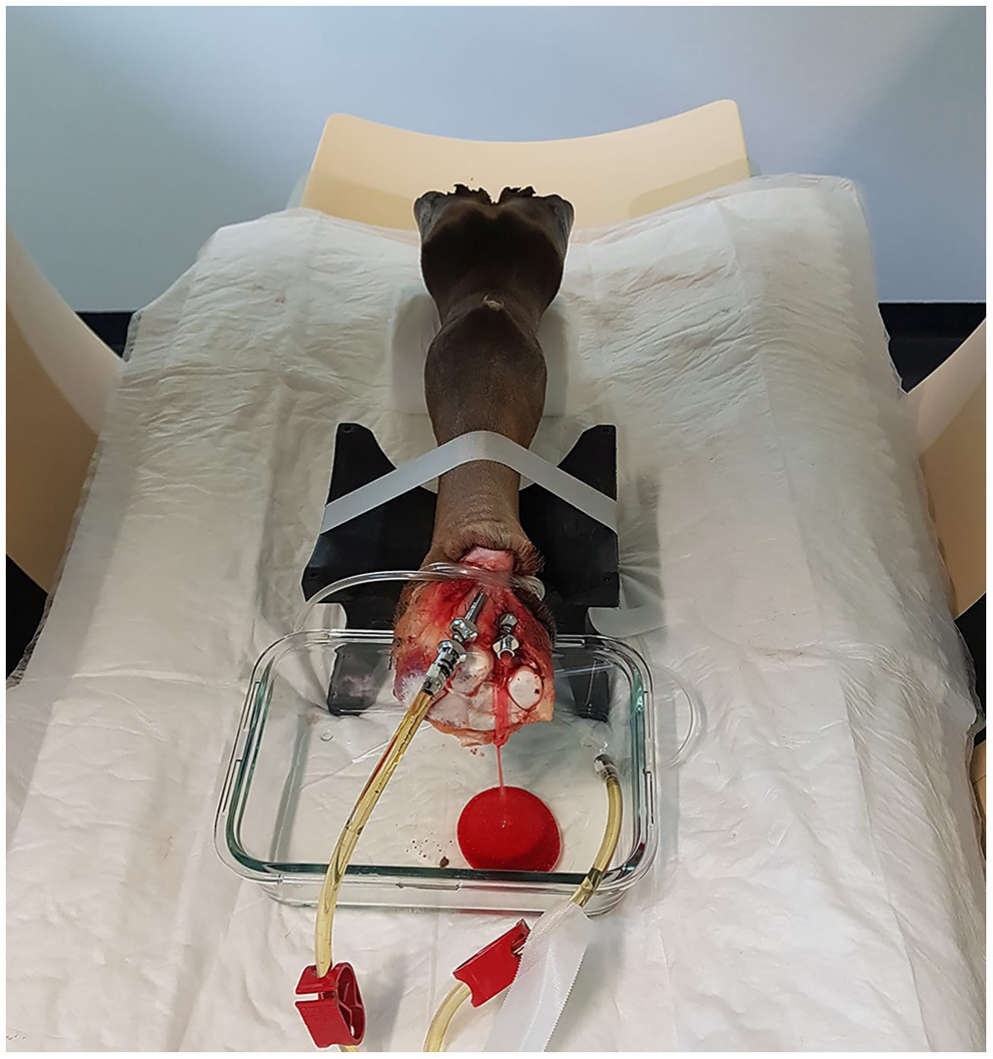
Positioning of the limb within the CT-gantry using two stands and a glass container to collect exiting perfusate. For this scan, only the left tube of the perfusion system was connected to the radial vein, allowing drainage of blood and contrast agent through the catheter of the median artery.

After thawing the limbs for ~24 h at room temperature, the feet were cleaned, and the hair clipped. Subsequently, two cannulas (18 G and 10 G) were inserted into the median artery and radial vein at the level of the disarticulation and secured with ligatures. A tourniquet was placed at the level of the cannulas, encircling most of the tissues at the cut end of the limb. The limb was placed in the CT scanner with its dorsal surface on two stands, a rubber stand at the level of the proximal metacarpus and one created from a rolled-up disposable underpad at the level of the pastern. A glass container was positioned under the proximal end of the limb to collect draining fluids during perfusion (Figure 1).

A native CT scan was acquired extending from just proximal to the proximal sesamoid bones to the distal-most aspect of the hoof, using parameters adapted from Van Hamel and colleagues (23): 140 kV, 200 mA, 2 mm slice thickness, 1 mm increment and a pitch of 0.688. Subsequently perfusion was performed in three phases, based on the MPMCTA-protocol (19): (1) perfusion through the median artery (100 mL/min for 110 s), (2) perfusion through the radial vein (100 mL/min for 50 s), and (3) arterial-venous combined (AVC) dynamic perfusion through both vessels simultaneously (100 mL/min for 30 s plus the duration of the scan). This protocol uses a volume of ~360 mL contrast medium per limb. Images were acquired in all three phases using the same parameters as for the native scan. During the arterial and venous phase scans, the perfusion was stopped just before the scanning process, whereas the AVC dynamic phase scans were performed during simultaneous arterial and venous injection of the contrast agent.

One limb (limb 6) was perfused with diluted hydrosoluble contrast medium through the median artery using the same settings as stated above (100 mL/min for 110 s). The experiment was stopped without performing the venous or AVC dynamic perfusion in this limb, as the scans obtained in the arterial phase already showed such marked extravasation it would have made the evaluation of further scans impossible.

**Figure 2:**
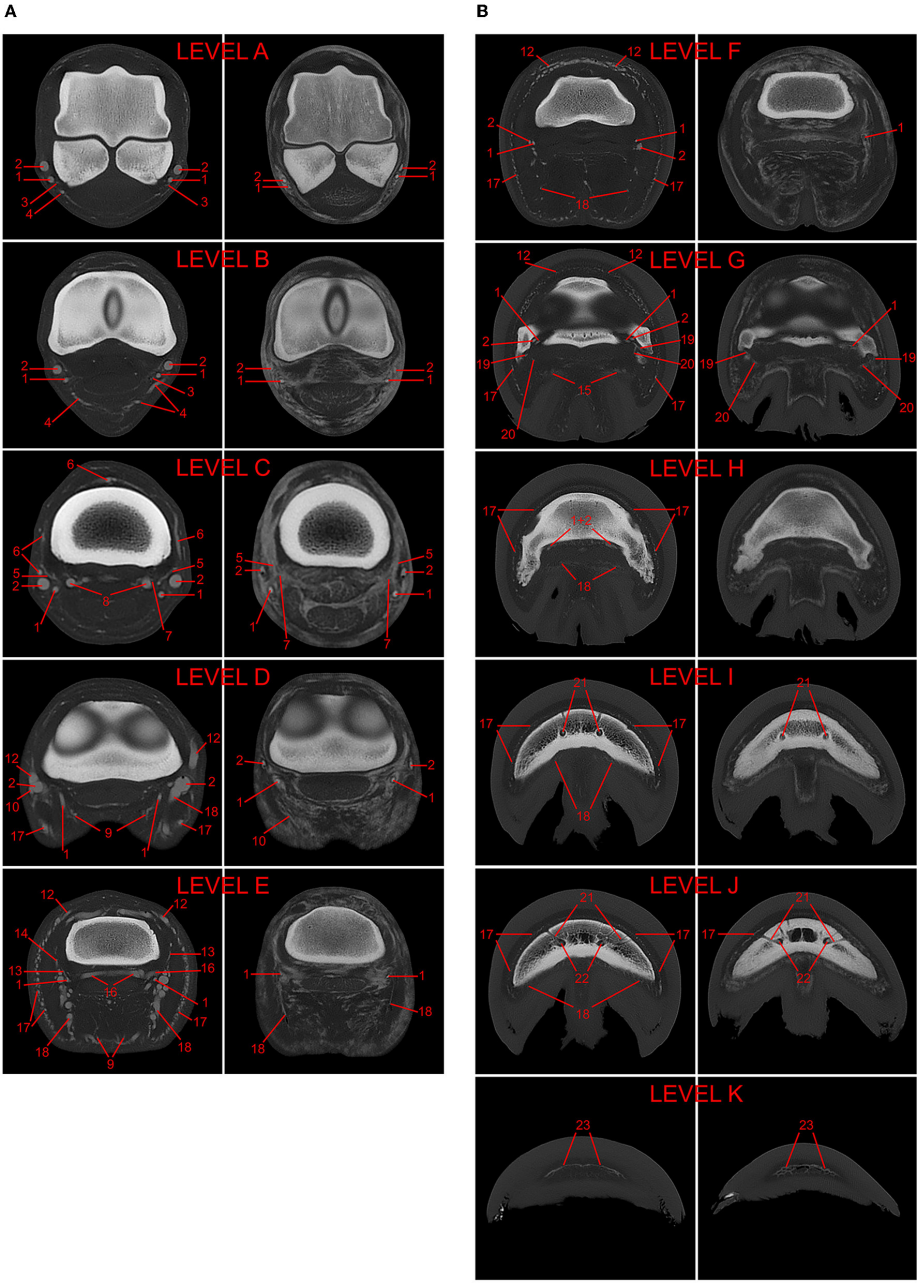
**(A)** 3D-reconstructed post-mortem CT-angiography of an equine distal forelimb after perfusion with a lipophilic contrast agent. The 11 evaluated levels are marked A–K in this reconstruction. **(B)** Comparison of paired transverse CT images in a bone algorithm (WW/WL: 800/2,000) at each of the 11 evaluated levels (A–K) from post-mortem CT-angiographic studies of two equine distal forelimbs perfused with either lipophilic (LP; left images; limb 1) or hydrosoluble (HS; right images; limb 6) contrast medium. The following vessels were evaluated: 1, palmar digital artery; 2, palmar digital vein; 3, arterial branch of the ergot; 4, venous branch of the ergot; 5, arterial dorsal branch of the proximal phalanx; 6, venous dorsal branch of the proximal phalanx; 7, arterial palmar branch of the proximal phalanx; 8, venous palmar branch of the proximal phalanx; 9, arterial branch of the digital torus; 10, venous branch of the digital torus; 11*, arterial coronary vessel; 12, venous coronary vessels; 13*, arterial dorsal branch of the middle phalanx; 14, venous dorsal branch of the middle phalanx; 15, arterial palmar branch of the middle phalanx; 16, venous palmar branch of the middle phalanx; 17, venous dermal plexus; 18, venous ungular plexus; 19, arterial dorsal branch of the distal phalanx; 20, arterial palmar branch of the distal phalanx; 21, terminal arch; 22, perforating branches; 23, circumflex vessels. *vessels not visible on the displayed transverse images.

The acquired images were evaluated by a board-certified veterinary radiologist (EVdV) and a veterinary doctoral student (CB) in consensus for visualization of the vasculature, presence of artifacts (i.e., filling defects and extravasation) and anatomic variations with the assistance of a veterinary anatomist (SK) where needed. Transverse images were evaluated at 11 specified levels (A-K), as described in the study of Collins and colleagues (9) and shown in Figure 2. Briefly, these regions consisted of the middle aspect of the proximal sesamoid bones (A), the metacarpophalangeal joint (B), the middle aspect of the proximal phalanx (C), the proximal interphalangeal joint (D), the proximal aspect of the middle phalanx (E), the coronary band (F), the distal interphalangeal joint (G), the proximal aspect of the distal phalanx (H), the middle aspect of the distal phalanx (I), the distal aspect of the distal phalanx (J), and the toe (K). The following vessels were assessed at their respective anatomical level: palmar digital arteries and veins (*A. et V. digitalis palmaris medialis/lateralis*; levels A–H), their palmar and dorsal branches of the proximal, middle and distal phalanx, respectively (*Rr. palmares/dorsales phalangis proximalis/mediae/distalis*; levels C, E, and G–H respectively), branch of the ergot (*Rr. tori metacarpei*; level B), branch of the digital torus (*A. et V. tori digitalis*; levels D–E and D, respectively), coronary vessels [*A. coronalis et Vv. coronales*; level D (artery) and levels D–G (veins)], dermal plexus (*Plexus dermalis*; levels E–I), ungular plexus (*Plexus ungularis*; levels E–I), terminal arch (*Arcus terminalis*; levels I–J), proximal and distal perforating branches (*Rr. perforantes proximales/distales*; level J), and circumflex vessels (*A. et V. marginis solearis*; level K).

The variables visualization (yes/no) and filling defects (complete/partial/none) were collected for the cross-sections of all the above-mentioned vessels at the specified levels. Extravasation (yes/no) was noted in each level, irrespective of the vessel cross-sections. Results are expressed as percentages for each of the following cohorts: arterial phase scans, venous phase scans, AVC dynamic phase scans, and hydrosoluble contrast medium perfusion scans. Additionally, percentages of all variables were calculated for each vessel (including all evaluated cross-sections). Differences between the cohorts were assessed using a Chi-squared test of independence. A *p*-value of <0.05 was considered statistically significant.

## Results

### Preparation and Perfusion

The 24 h thawing period was sufficient to defrost all limbs, and the median artery and radial vein were easy to identify and cannulate. The application of a tourniquet at the level of the cannulas decreased the leakage of contrast agent from the disarticulated surface of the limbs during perfusion. Perfusion using a roller pump was effective and the perfusate, mixed with blood, was observed exiting the limb through larger vessels and the cut surface within 20 s of beginning the perfusion. The overall scan time, from the start of arterial perfusion to the end of the last scan, was between 10 and 22 min.

### Visualization of the Vasculature

In limbs reperfused with the lipophilic contrast agent, overall 93.1% of the evaluated vessel cross-sections were identified combining all scans and phases. The number of vessel cross-sections visualized at every level for each limb is listed in Table 1 for the arterial, venous and AVC dynamic phases. The visibility of the evaluated vascular structures at each level is reported in Table 2. This table shows the visibilities for all limbs perfused with lipophilic contrast combined, for each of the 3 phases and for all phases combined. The main vessels, i.e., medial and lateral digital veins (100%), terminal arch (100%), perforating branches (100%), and circumflex vessels (100%) were consistently visualized in all limbs, at all expected levels and in all phases. Smaller caliber vessels, especially the coronary arteries (77%) and the arterial branches of the ergot (77%), of the digital torus (80%), and of the distal phalanx (73% dorsal, 52% palmar), were not as readily identified in all limbs when combining all scans and phases. Both venous plexuses and the digital arteries were visualized in nearly all limbs and all expected levels (>98%), and overall, the venous vasculature had a significantly (*p* < 0.00001) higher rate of visualization (97.9%) compared the arterial vasculature (84.7%).

**Table 1:** Comparison vascular visualization of different limbs perfused with lipophilic contrast.

	**limb 1**			**limb 2**			**limb 3**			**limb 4**			**limb 5**		
	**Art**	**Ven**	**Dyn**	**Art**	**Ven**	**Dyn**	**Art**	**Ven**	**Dyn**	**Art**	**Ven**	**Dyn**	**Art**	**Ven**	**Dyn**
**Level A** (*n =* 4)	4	4	4	4	4	4	4	4	4	4	4	4	4	4	4
**Level B** (*n =* 8)	8	8	8	5	6	6	8	8	8	7	7	7	8	8	8
**Level C** (*n =* 12)	12	12	12	9	12	11	12	12	12	11	11	11	12	12	12
**Level D** (*n =* 10)	8	8	8	8	8	8	10	10	10	9	9	9	9	9	9
**Level E** (*n =* 22)	20	21	22	12	15	16	21	19	20	22	22	22	22	22	22
**Level F** (*n =* 10)	10	10	10	9	10	10	10	10	10	10	9	10	8	8	10
**Level G** (*n =* 14)	12	12	14	8	12	12	13	13	13	12	12	12	13	14	14
**Level H** (*n =* 12)	7	7	8	7	7	7	10	10	10	10	10	11	7	7	10
**Level I** (*n =* 6)	6	6	6	4	4	6	6	6	6	6	6	6	6	6	6
**Level J** (*n =* 6)	6	6	6	6	6	6	6	6	6	6	6	6	6	6	6
**Level K** (*n =* 2)	2	2	2	2	2	2	2	2	2	2	2	2	2	2	2

*This table lists the number of vessel cross-sections visualized at every level for each limb (1–5) and this for the arterial (art), venous (ven) and arterial-venous combined dynamic (dyn) phases. The number of expected vessel cross-sections (medial + lateral) for every level is reported below the respective levels in the first column*.

**Table 2:** Comparison of vascular visualization at every level for all limbs perfused with lipophilic contrast combined, both per phase and for all phases combined.

	**Limbs 1–5**	**%**	**Limbs 1–5**	**%**	**Limbs 1–5**	**%**	**Limbs 1–5**	**%**
	**A Vi** **(*n =* 10)**	**A Vi%**	**V Vi** **(*n =* 10)**	**V Vi%**	**D Vi** **(*n =* 10)**	**D Vi%**	**C Vi** **(*n =* 30)**	**C Vi%**
**Level A**								
**A. digitalis palmaris**	**10**	**100**	**10**	**100**	**10**	**100**	**30**	**100.0**
**V. digitalis palmaris**	**10**	**100**	**10**	**100**	**10**	**100**	**30**	**100.0**
**Level B**								
**A. digitalis palmaris**	**9**	**90**	**10**	**100**	**10**	**100**	**29**	**96.7**
ramus of the ergot	7	70	8	80	8	80	23	76.7
**V. digitalis palmaris**	**10**	**100**	**10**	**100**	**10**	**100**	**30**	**100.0**
ramus of the ergot	10	100	10	100	10	100	30	100
**Level C**								
**A. digitalis palmaris**	**10**	**100**	**10**	**100**	**10**	**100**	**30**	**100.0**
R. dorsalis phalangis proximalis	9	90	10	100	10	100	29	96.7
R. palmaris phalangis proximalis	8	80	10	100	9	90	27	90.0
**V. digitalis palmaris**	**10**	**100**	**10**	**100**	**10**	**100**	**30**	**100.0**
R. dorsalis phalangis proximalis	9	90	9	90	10	100	28	93.3
R. palmaris phalangis proximalis	10	100	10	100	10	100	30	100.0
**Level D**								
**A. digitalis palmaris**	**10**	**100**	**10**	**100**	**10**	**100**	**30**	**100.0**
A. tori digitalis	8	80	8	80	8	80	24	80.0
**V. digitalis palmaris**	**10**	**100**	**10**	**100**	**10**	**100**	**30**	**100.0**
V. tori digitalis	8	80	9	90	9	90	26	86.7
Vv. coronales	9	90	10	100	10	100	29	96.7
**Level E**								
**A. digitalis palmaris**	**10**	**100**	**10**	**100**	**10**	**100**	**30**	**100.0**
A. tori digitalis	8	80	8	80	8	80	24	80.0
A. coronalis	8	80	8	80	7	70	23	76.7
A. dorsalis phalangis mediae	8	80	8	80	10	100	26	86.7
A. palmaris phalangis mediae	7	70	6	60	8	80	21	70.0
**V. digitalis palmaris**	**10**	**100**	**10**	**100**	**10**	**100**	**30**	**100.0**
Plexus dermalis	10	100	10	100	10	100	30	100.0
Plexus ungularis	10	100	10	100	10	100	30	100.0
Vv. coronales	10	100	10	100	9	90	29	96.7
V. dorsalis phalangis mediae	10	100	10	100	10	100	30	100.0
V. palmaris phalangis mediae	10	100	10	100	10	100	30	100.0
**Level F**								
**A. digitalis palmaris**	**9**	**90**	**10**	**100**	**10**	**100**	**29**	**96.7**
**V. digitalis palmaris**	**10**	**100**	**10**	**100**	**10**	**100**	**30**	**100.0**
Plexus dermalis	9	90	6	60	10	100	25	83.3
Plexus ungularis	10	100	10	100	10	100	30	100.0
Vv. coronales	10	100	10	100	10	100	30	100.0
**Level G**								
**A. digitalis palmaris**	**8**	**80**	**10**	**100**	**10**	**100**	**28**	**93.3**
R. dorsalis phalangis distalis	8	80	10	100	10	100	28	93.3
R. palmaris phalangis distalis	5	50	6	60	6	60	17	56.7
**V. digitalis palmaris**	**10**	**100**	**10**	**100**	**10**	**100**	**30**	**100.0**
Plexus dermalis	10	100	10	100	10	100	30	100.0
Plexus ungularis	9	90	10	100	10	100	29	96.7
Vv. coronales	10	100	10	100	10	100	30	100.0
**Level H**								
**A. digitalis palmaris**	**10**	**100**	**10**	**100**	**10**	**100**	**30**	**100.0**
R. dorsalis phalangis distalis	5	50	5	50	6	60	16	53.3
R. palmaris phalangis distalis	4	40	4	40	6	60	14	46.7
**V. digitalis palmaris**	**10**	**100**	**10**	**100**	**10**	**100**	**30**	**100.0**
Plexus dermalis	9	90	9	90	10	100	28	93.3
Plexus ungularis	8	80	10	100	10	100	28	93.3
**Level I**								
**Arcus terminalis/Arcus terminalis**	**10**	**100**	**10**	**100**	**10**	**100**	**30**	**100.0**
Plexus dermalis	10	100	10	100	10	100	30	100.0
Plexus ungularis	10	100	10	100	10	100	30	100.0
**Level J**								
**Arcus terminalis/Arcus terminalis**	**10**	**100**	**10**	**100**	**10**	**100**	**30**	**100.0**
Rr. perforantes proximales	10	100	10	100	10	100	30	100.0
Rr. perforantes distales	10	100	10	100	10	100	30	100.0
**Level K**								
**A. (V.) marginis solearis**	**10**	**100**	**10**	**100**	**10**	**100**	**30**	**100.0**

*This table lists the combined number of vessel cross-sections visualized of every evaluated vascular structure for all 11 evaluated levels (A-K) of all limbs perfused with lipophilic contrast agent (limbs 1–5). The number of visible vessel cross sections are mentioned both as absolute values and as percentages of the expected number of vessel cross-sections, and this for the arterial phase (A Vi; A Vi%), venous phase (V Vi; V Vi%), the arterial-venous combined dynamic phase (D Vi; D Vi%) and for all phases combined (C Vi; C VI%). The main vascular structures for each level are mentioned in bold, to differentiate them from their branches. The number of expected vessel cross-sections (medial + lateral) for each evaluated vascular structure for all 5 limbs combined, is mentioned below each phase (n = 10) and for the 3 phases combined (n = 30). In case of anatomical variations, vessel cross-sections that were not visualized their expected level, but at another level (1 more proximal for all), were added to the number of the expected level for clarity of the table*.

Overall visualization rates among the individual limbs was fairly consistent (91.5–95.3%) with the exception of limb 2, which allowed for a lower number of identified vessel cross-sections (78.0%).

### Artifacts

Diffuse extravasation was noted in two of the five limbs reperfused with Angiofil^®^: limb 2, the limb that underwent two freezing-thawing cycles, and limb 4. In both cases, contrast medium could be seen in the connective tissue surrounding the vasculature as well as inside the tendons and ligaments. No vessel defects could be identified on the CT scans as the origin for the leaking contrast medium. Extravasation was more severe proximally, affecting levels A-H in both limbs.

Overall, filling defects were noted in 17.4% of all visible vessel cross-sections, ranging in individual limbs from 12.4 to 23.1%. Although filling defects occurred most commonly in the arterial branch of the ergot (69.6%), overall, they were more often observed in the venous vasculature (23.2%) than in the arterial vasculature (9.5%). The venous plexuses showed a highly variable degree of filling defects (0-100%). For all phases combined, filling defects were most commonly observed in the dermal vessels at levels H (57.1%) and I (80%) and in the ungular vessels at level F (53%) and H (35.7%).

### Comparison of Phases

**Figure 3:**
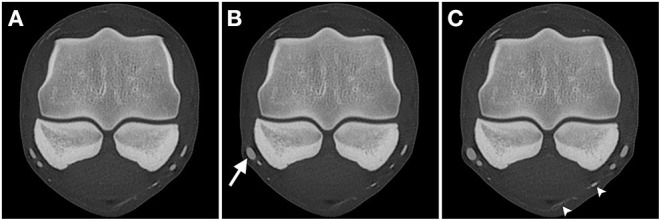
Transverse CT images of limb 3 in a bone algorithm (WW/WL: 800/2,000), showing the comparison of vessel-visualization in the **(A)** arterial, **(B)** venous, and **(C)** arterial-venous combined (AVC) dynamic phase scans obtained at the level of the middle aspect of the proximal sesamoid bones (level A). The digital arteries and veins are markedly flattened in the arterial **(A)** and venous **(B)** phase, with only the lateral palmar digital vein being more filled on the venous phase (arrow). In the AVC dynamic phase **(C)** all vessels appear larger and the medial venous branch of the ergot (arrowheads) is more easily identified. Top of the image is dorsal, left is lateral.

AVC dynamic perfusion resulted in the highest degree of visualization with 95.1% of all expected vessel cross-sections being identified. The first (arterial) phase of perfusion resulted in a significantly lower (*p* < 0.05) lower visualization rate (90.1%), while the venous phase resulted in an intermediate visualization rate of 93.2%. As for the overall visualization, the arterial vasculature had a significantly lower rate of visualization than the venous vasculature in the arterial (80.5% vs. 96.8%), the venous (85.5% vs. 97.5%) and the AVC dynamic phases (88% vs. 99.3%). Additionally, in the AVC dynamic phase the individual vessel cross-sections appeared more filled (larger and more rounded), making them subjectively easier to identify (Figure 3).

**Figure 4:**
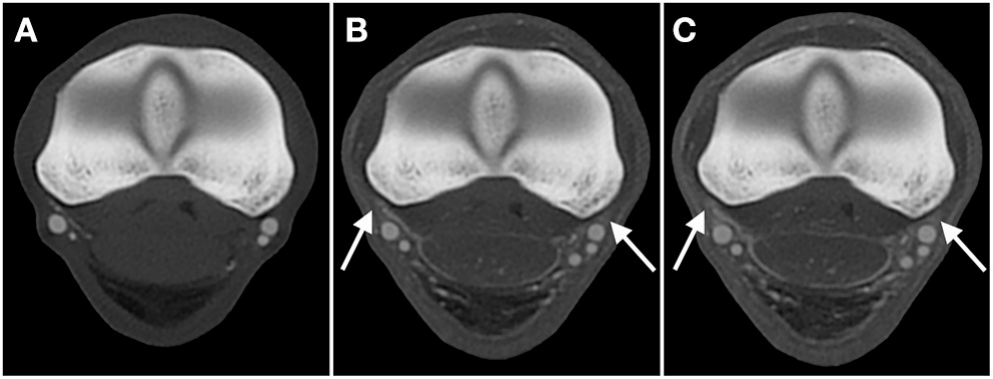
Transverse CT images in a bone algorithm (WW/WL: 800/2,000), showing the comparison of extravasation in the **(A)** arterial, **(B)** venous, and **(C)** arterial-venous combined dynamic phase scans obtained of limb 2, the limb which underwent two freezing-thawing cycles, at the level of the metacarpophalangeal joint (level B). While progressive increased extravasation in this limb is evident (arrows; B, C), decreasing the subjective quality of the scans, the degree of extravasation with lipophilic contrast remains clearly lower when compared to the limb perfused with hydrosoluble contrast in Figure 2. Top of the image is dorsal, left is lateral.

While visualization improved in later phases, extravasation worsened in the two limbs where it was present. In fact, limb 2 only showed considerable extravasation in the AVC dynamic phase. Larger amounts of extravasation impeded the exact delineation of some vessels, which considerably deteriorated the image quality upon subjective assessment (Figure 4).

Filling defects appeared in similar rates during the arterial (19.4% of all evaluated vessel cross-sections) and venous (22.5%) phase but decreased significantly (*p* < 0.0001) in dynamically perfused vasculature (10.2%).

### Comparison to Hydrosoluble Contrast Agent

In this study, one limb (limb 6, Figure 2) was perfused with a diluted iodinated contrast agent commonly used for *in vivo* CT-angiography. Vessel visualization was significantly (*p* < 0.00001) reduced in this limb, with 74.0% identifiable vessel cross-sections compared to the 90.1% achieved during the arterial phase of perfusion with lipophilic contrast agent.

Both types of artifacts, extravasation and filling defects, occurred significantly more often in the vasculature perfused with the hydrosoluble contrast agent. Filling defects were found in 52.6% of all evaluated vessel cross-sections, which is a significant (*p* < 0.00001) increase compared to the limbs perfused with lipophilic contrast medium. Additionally, the observed generalized extravasation considerably reduced the subjective image quality.

### Anatomic Variations

The major vascular structures as well as their branches were consistent in their location and size between the individual specimens. The observed slight variations concerned the proximodistal level of branching. In two limbs each, the coronal arteries or veins bilaterally branched off one level proximal to the expected location. No considerable differences were found between left and right forelimbs.

One unusual anatomic variation occurred in limb 6, which consisted of the digital artery and vein switching places (i.e., the digital artery being located dorsal to the digital vein) proximal to the metacarpophalangeal joint over a length of ~10 cm.

## Discussion

### Preparation and Perfusion

The MPMCTA protocol used in this study proved to be successful, allowing the reperfusion and subsequent 3D-visualization of the vasculature in frozen-thawed equine distal limbs without prior preparation. With this method, equine cadaver limbs can be used as a model for future vascular research which minimizes both the personnel and animal exposure to radiation and the need for experimental animals, thus honoring the ALARA and 3R principles.

Angiofil^®^, a lipophilic contrast agent consisting of iodized linseed oil (19), was chosen as a contrast agent in this study. It was developed specifically for post-mortem CT-angiography and has been used without prior sample preparation (17, 18) in frozen-thawed human cadavers up to 4 months after death (19) and even a seventeenth-century mummy (24).

Perfusion was performed with a simple roller pump as a low-cost and accessible alternative to more sophisticated and finely adjustable pumps, such as specifically developed post-mortem perfusion pumps (18, 19, 24). The roller pump allowed good reperfusion of both arteries and veins, supporting the findings of other studies that have achieved sufficient perfusion with basic roller pumps (17), immersion pumps (25) or manual injection (26).

### Visualization of the Vasculature

Visualization of the vasculature was excellent in most cases, specifically of the main vessels and venous branches. The lower visualization of smaller arterial branches coincided with the presence of extravasation in 21 of the 22 not visible vessel cross-sections. Thus, the low visualization rate is likely due to extravasated contrast agent obscuring vessels with a small diameter. It is conceivable that a higher pressure or longer perfusion duration could increase the size and thus visibility of these vessels despite the presence of extravasation. However, the optimal perfusion settings for post-mortem angiography seem to be a matter of controversy (20) and further studies are needed to explore their effect on the perfusion of frozen-thawed equine distal limbs.

Medial, lateral, dorsal and palmar vessels were all identified at similar rates (in total 742 vessel cross-sections medially and 743 laterally were visualized). In previous angiographic studies of the distal limbs of horses under general anesthesia, differences in perfusion of the recumbent and non-recumbent side were observed (9). To overcome compression of the mostly palmar located vasculature, all limbs were placed in supine position. While this proved to be successful for post-mortem perfusion, this position cannot be obtained *in vivo*. Therefore, the effect of positioning on filling and visualization of the vessels should be evaluated further with post-mortem angiographic studies of equine distal limbs.

### Artifacts

Overall, extravasation and filling defects, were uncommon, affecting 23% of all evaluated levels and 17.4% of all visible vessels cross-sections. In fact, extravasation was only noted in two of the limbs perfused with Angiofil^®^, one of which (limb 2) had been frozen and thawed twice before perfusion. It is likely that this process accelerated decomposition, which increased the permeability of vascular walls and made the limb more susceptible to extravasation (17, 21). This advanced decomposition may also explain the reduced rate of visualization in limb 2. Therefore, the use of limbs that underwent 2 freezing-thawing cycles should be avoided for post-mortem angiography studies. The exact cause for the observed extravasation in limb 4 is unknown. The authors hypothesize that ante-mortem pathological mechanisms that caused increased vascular permeability are likely, as the limb underwent an identical protocol to the other 3 limbs that did not show any extravasation. Possible mechanisms include septic (27), endotoxic (28, 29), and inflammatory vasculitis (30). Endotoxemia can be caused by common equine gastro-intestinal diseases like colitis and severe colic (31). Injuries to local structures like the flexor tendons may also lead to vascular damage, but no tendon alterations were observed on external inspection nor radiologically (32, 33). Since no medical history on this horse was available, the reasons for the noted extravasation remain speculative. A longer duration of freezing is considered an unlikely cause as we have perfused limbs frozen for more than 4 months without any signs of extravasation (unpublished data).

Many of the observed filling defects in the larger vessels consisted of small gas bubbles and did not seem to occlude the vascular lumen. It was impossible to avoid the infiltration of air through the cut end of the vessels while handling the disarticulated limbs and it is highly likely that this air got forced deeper into the vasculature during perfusion and subsequently formed visible gas bubbles. However, exact differentiation between soft tissue (blood) and gas attenuating filling defects was not made in this study. For multiple small filling defects, specifically in smaller vessels, partial volume averaging artifacts prevented the differentiation between both types of filling defects. The highest rate of filling defects was found in the arterial branches of the ergot, although the venous vasculature showed higher rates overall. It is unclear if these findings are coincidental, but this will likely become apparent when this technique is performed in more limbs.

### Comparison of Phases

In the present study, visualization of both arterial and venous vasculature was achieved in all three phases of perfusion. This is in contrast to the results reported in human forensic medicine, where the arterial and venous phases are designed to expand the arterial or venous systems, respectively (19, 34). A possible explanation for this disparity is the high number of arteriovenous shunts in the equine limb (35, 36) allowing for efficient filling of the venous side during arterial perfusion. Additionally, while the human MPMCTA-protocol suggests longer venous than arterial perfusion (19), perfusion duration in this study was based on the time needed for contrast material to be observed exiting the limb, which was longer for the first (arterial) phase. It is possible that shorter perfusion times during the arterial phase may have caused less filling of the veins, however this was not considered a limitation for the visualization of the arteries.

Images obtained during AVC dynamic perfusion not only showed objectively higher rates of visualization and lower rates of filling defects, but also improved the quality upon subjective assessment. The vasculature appeared more filled, in cross-section, which made specifically the smaller vessels easier to be identified. Therefore, insufficient vascular filling could be interpreted as an additional artifact, a flattening artifact, which should be evaluated in future post-mortem angiography studies. These findings are in accordance with human studies reporting the most effective filling of the vasculature system, and thus best image quality, during the AVC dynamic phase (19).

However, in limbs where extravasation was present, it increased during the later phases and was most pronounced in the AVC dynamic phase. This finding may be explained by the increased overall time of perfusion, the increased intravascular pressure during AVC dynamic perfusion, or a combination of both. Specifically, the simultaneous arterial and venous perfusion was an adaption made to the original human protocol where only the arterial side was injected during the dynamic phase, leaving the venous side open for drainage. While this simultaneous arterial-venous perfusion was performed to increase intravascular pressure, which increased the likelihood to perfuse also the smaller vessels in the foot, this likely also caused a higher risk for extravasation of contrast. It may be possible to achieve similarly high-quality images while minimizing extravasation due to prolonged perfusion by directly performing the AVC dynamic phase with simultaneous arterial and venous perfusion, skipping the separate arterial and venous phases. This would also allow for a more time- and material-efficient protocol. However, its effects on the quality of the vascular visualization needs to be examined.

### Comparison to Hydrosoluble Contrast Agent

Lipophilic contrast agents are preferably used for post-mortem perfusion to minimize extravasation and to flush out blood clots, thus overcoming the two main limitations of post-mortem angiography (18, 20). In our study, the limb perfused with hydrosoluble contrast (limb 6) showed a high degree of generalized extravasation, reducing subjective image quality and vessel visualization. Also filling defects were identified more frequently, however no evidence of blood clots obstructing the perfusion was found as even some small-caliber vessels in the most distal aspect of the foot were successfully visualized. Nevertheless, the generalized extravasation observed after just one perfusion phase demonstrates the impracticality of using hydrosoluble contrast agents in post-mortem angiography.

### Anatomic Variations

The main anatomy of the vasculature in the evaluated forelimbs was consistent, independent of the side or laterality of the vessels. However, there were a few slight variations concerning the level at which some smaller venous and arterial branches originated. Remarkably, in one limb a focal switch in relative positions of the digital artery and vein occurred at the level of the metacarpophalangeal joint.

Overall, these findings support previous studies reporting only minor variations in the localization of the branches of the major vessels (7, 9).

### Limitations and Future Research

This study has a few limitations. No macroscopic and/or histopathologic examination was performed after the post-mortem perfusion of the cadaver limbs. A histological comparison of the vascular structures between the limbs could have identified a cause for the unexpected extravasation observed in limb 4 and confirmed the more advanced decomposition of the vascular walls in limb 2 and would be of benefit for future studies. The lack of comparison between the visualization of the vessels with this post-mortem perfusion technique and clinical angiography studies performed in alive patients using power injectors could be considered an additional limitation. However, the aim of this study was to prove feasibility of restoring vascular flow in frozen-thawed equine distal limbs without any prior preparation using a procedure adapted from the human MPMCTA protocol. The visualization of the different vessels was reported only to support this hypothesis and a comparison with *in vivo* angiography results was not within the scope of this study. However, detailed comparison of ante- and post-mortem angiography of the equine distal limb using optimized protocols would be an interesting future study and provide a validation for the use of frozen-thawed cadaver limbs as an equine vascular model.

Currently MPMCTA is mainly extensively used and described in forensic human medicine as a complementary technique to autopsy because it allows observer-independent, reproducible and minimally invasive assessment of the vascular system (34, 37, 38). It enables the screening for vascular lesions that would be difficult or impossible to detect during autopsy and allows for a later focused dissection of the target area for macroscopic or histological confirmation of disease (37, 39). It would be interesting to test the value of full body MPMCTA in animal species such as dogs and cats, as well as new-world camelids or foals and calves. Its use in adult larger animals such as horses or cows would require large volumes of contrast and is restricted by the currently limited availability of large-bore veterinary CT scanners. As an alternative, future studies may investigate the application of optimized versions of the protocol described in this study as selective post-mortem CT angiography on separated body parts, as these would fit in most standard multi-detector CT scanners, which are more commonly available. If feasible, these animal post-mortem CT angiographies may have similar benefits as mentioned above for human forensic studies, serving as an aid to standard autopsy in determining the presence and location of vascular disease such as vascular malformations, lacerations or thrombi, but also providing detailed and 3D documentation of lesions for insurance or legal purposes eg. after fatal (bite) accidents or severe mutilation. Although studies on completely virtual autopsies using full body CTs (virtopsy) have been described in veterinary medicine as an alternative option to investigate the cause of death whenever a full autopsy is not possible or desired, their use is still uncommon (40). Adding angiography to the current virtopsy protocols may help to overcome some of the current limitations such as poor vascular visualization. Future studies need to confirm both the feasibility and the value of partial and full body MPMCTA in animals. Although MPMCTA for veterinary medicine has not yet been described, post-mortem angiography using Angiofil^®^ has already been applied for vascular studies in much smaller species, such as mice (22). That study visualized the vascular system of mice down to the level of the renal capillaries using microcomputed tomographic scans. If future studies confirm that similarly sized blood vessels can be visualized in the equine distal limb, this model may show the vascular effects of various therapies and angiogenesis three dimensionally allowing more specific histologic examinations, in studies targeting for example tendon lesions or laminitis.

## Conclusion

Frozen-thawed equine distal forelimbs without prior preparation can be successfully reperfused with a lipophilic contrast agent using a flow rate of 100 mL/min in three consecutive phases (arterial, venous, and AVC dynamic) with fixed perfusion durations (110, 50, and 30 s, respectively). This protocol allows for a high-quality 3D-visualization and detailed evaluation of the vascular anatomy *in situ* with CT-imaging, while honoring both the 3R and ALARA principles. Further studies are needed to optimize the technique, maintaining the good visualization while reducing the risk of extravasation to a minimum.

Therefore, establishing a standardized protocol for high-quality multi-phase post-mortem CT-angiography in equine distal limbs should be the first aim for future studies using this technique.

## Data Availability Statement

The raw data supporting the conclusions of this article will be made available by the authors, upon request.

## Ethics Statement

This research was performed on cadaver parts of animals slaughtered or euthanized for reasons unrelated to this study and therefore exempt from ethical review according to the guidelines from the Kanton of Bern, Switzerland. Written informed consent was obtained from the owners for the participation of their animals in this study.

## Author Contributions

EVdV and DS initiated the project. CB and EVdV contributed to all parts of the project, participated in purchasing, organizing the materials necessary to the project, preparing and realizing the practical cadaveric part of the project, participated in data acquisition, data analysis, interpretation, and creation of the figures. CB prepared the first draft of the manuscript. SG contributed with advice for post-mortem angiography perfusion. All authors critically revised the manuscript for important intellectual content, read, and approved the final version of the article.

## Funding

This work was supported by the Stiftung Tierspital Bern, Department of Clinical Veterinary Science, Bern University, Switzerland.

## Conflict of Interest

The authors declare that the research was conducted in the absence of any commercial or financial relationships that could be construed as a potential conflict of interest.

## Publisher's Note

All claims expressed in this article are solely those of the authors and do not necessarily represent those of their affiliated organizations, or those of the publisher, the editors and the reviewers. Any product that may be evaluated in this article, or claim that may be made by its manufacturer, is not guaranteed or endorsed by the publisher.
